# PMCA-replicated PrP^D^ in urine of vCJD patients maintains infectivity and strain characteristics of brain PrP^D^: Transmission study

**DOI:** 10.1038/s41598-019-41694-0

**Published:** 2019-03-26

**Authors:** Ignazio Cali, Jody Lavrich, Fabio Moda, Diane Kofskey, Satish Kumar Nemani, Brian Appleby, Fabrizio Tagliavini, Claudio Soto, Pierluigi Gambetti, Silvio Notari

**Affiliations:** 1 Case Western Reserve University, Department of Pathology, Cleveland, Ohio, USA; 2 Fondazione IRCCS Istituto Neurologico Carlo Besta, Neurology 5 and Neuropathology Unit, Milano, Italy; 3 Case Western Reserve University, National Prion Disease Pathology Surveillance Center and Departments of Neurology and Psychiatry, Cleveland, Ohio, USA; 4 University of Texas Health Science Center, Department of Neurology, Houston, Texas USA

## Abstract

The presence of abnormal, disease-related prion protein (PrP^D^) has recently been demonstrated by protein misfolding cyclic amplification (PMCA) in urine of patients affected with variant Creutzfeldt-Jakob disease (vCJD), a prion disease typically acquired from consumption of prion contaminated bovine meat. The complexity and multistage process of urine excretion along with the obligatory use of PMCA raise the issue of whether strain characteristics of the PrP^D^ present in vCJD brains, such as infectivity and phenotype determination, are maintained in urine excreted PrP^D^ and following amplification by PMCA. We inoculated transgenic mice expressing normal human PrP with amplified urine and brain homogenate achieving the same 100% attack rate, similar incubation periods (in both cases extremely long) and histopathological features as for type and severity of the lesions. Furthermore, PrP^D^ characteristics analyzed by immunoblot and conformational stability immunoassay were indistinguishable. Inoculation of raw vCJD urine caused no disease, confirming the extremely low concentration of PrP^D^ in vCJD urine. These findings show that strain characteristics of vCJD brain PrP^D^, including infectivity, are preserved in PrP^D^ present in urine and are faithfully amplified by means of PMCA; moreover, they suggest that the PrP^D^ urine test might allow for the diagnosis and identification of disease subtype also in sporadic CJD.

## Introduction

The diversity of human prion diseases is in part due to the presence of three etiological forms – sporadic, inherited and acquired by infection – while in all other neurodegenerative diseases only the sporadic and inherited forms are currently recognized^1,2^. Furthermore, available evidence points to the brain as the organ where prion diseases start in the sporadic and most inherited forms^2^. By contrast, the great majority of acquired human prion diseases including iatrogenic and variant (v) Creutzfeldt-Jakob disease (CJD), start in peripheral tissues and subsequently propagate to the central nervous system^3^. vCJD, a condition characteristically acquired by consumption of prion contaminated bovine meat^4,5^, is a case in point. In vCJD, conversion by the bovine prion of the host normal or cellular prion protein (PrP^C^) generating a human abnormal and disease-related PrP (PrP^D^) appears to occur in the gut-associated lymphoid tissues and to propagate to the brain following route(s) yet to be completely defined^6–8^. Many visceral organs and peripheral body fluids such as blood and urine may be exposed to prion infection during this process^3^. Indeed, in vCJD, the presence of proteinase K (PK)-resistant PrP^D^ (resPrP^D^) has been shown in several visceral organs, including adrenal gland, spleen, mesenteric lymph nodes, liver, pancreas and kidney^9,10^. Recently, we have demonstrated the presence of minute amounts of PrP^D^ in urine of vCJD patients^11^. Using protein misfolding cyclic amplification (PMCA), PrP^D^ was reliably detected affording an accurate and non-invasive diagnostic test of prion disease^11^. By contrast, demonstration of PrP^D^ in urine of patients with sCJD has been more challenging^11,12^.

The mechanisms leading to the presence of PrP^D^ in urine of vCJD patients remain to be determined^11,12^. The complex, multistage process of urine formation, raises the possibility that, while spreading to urine, PrP^D^ undergo subtle conformational changes altering strain characteristics. Moreover, this issue is further compounded by the extreme under-representation of PrP^D^ in urine that requires extensive amplification or enrichment procedures^11,12^.

Transmission to appropriate hosts has been shown to be a suitable approach to define and compare prion strain properties^13^. To this aim, we inoculated transgenic (Tg) mice expressing human PrP 129 M (Tg40) with PrP^D^ obtained from vCJD urine following PMCA and untreated vCJD brain homogenate (BH). Raw vCJD urine was injected as control. Mice injected with PMCA-treated products and those injected with untreated vCJD BH developed prion diseases that were phenotypically indistinguishable and were characterized by similar attack rates and extended incubation periods. Moreover, resPrP^D^ displayed similar conformational features. By contrast, none of the animals inoculated with untreated vCJD urine developed a prion disease.

These data have been partially presented at Prion 2018 (May 22–25, 2018; Santiago de Compostela, Spain).

## Results

### Transmission characteristics

Inoculation of Tg40 mice with PMCA-treated vCJD urine resulted in prion disease with an attack rate of 100% and incubation periods, as measured by days post inoculation (dpi), of 661 ± 45 and 713 ± 36, depending on the final dilution (1x or 1:10, respectively) of the inoculated PMCA product (Table 1). These results were comparable to those obtained with the transmission of vCJD BH where attack rate again was always 100% and incubation periods varied between 574 ± 104 and 648 ± 39 dpi, according to the 10% or 1% BH concentration of the inoculum (Table 1). The only statistically significant difference was observed when comparing dpi following inoculation of the more diluted PMCA-treated urine (1:10) to the dpi of the more concentrated vCJD BH (10%) inoculum (Table 1). Inoculation of PMCA-treated unseeded-substrate, as negative control, does not have any effect on mice life span. None of the 96 Tg40 mice inoculated with raw vCJD urine tested positive for prion disease up to 707 dpi.

**Table 1:** Transmission to Tg40 mice of PMCA-treated vCJD urine, PMCA negative control, untreated vCJD urine and vCJD brain homogenate (BH).

Inoculum	Histology & immunohist.		resPrP^D^ Western blot		
	Attack rate (posit./tot.)	Incubation periods (dpi)^c^	Attack rate (posit./tot.)	PrP^D^ type	Incubation periods (dpi)^c^
PMCA-treated vCJD urine^a^ (1x)^b^	5/5	679 ± 39	9/9	T2B^d^	661 ± 45
PMCA-treated vCJD urine (1:10)^b^	3/3	718 ± 21	8/8	T2B	713 ± 36*
PMCA of unseeded substrate	0/4	0/718 ± 18	0/9	—	0/673 ± 182
Raw vCJD urine	0/52	0/700 ± 179	0/96	—	0/707 ± 148
vCJD BH 10%	6/6	615 ± 78	9/9	T2B	574 ± 104*
vCJD BH 1%	2/2	657 ± 51	3/3	T2B	648 ± 39

^a^Six rounds of PMCA have been performed on the resuspended pellet derived from 1 ml urine. ^b^Inoculum 1x and 1:10 correspond to undiluted and to diluted 1:10, respectively. ^c^dpi values are express as mean ± standard deviation. ^d^T2B means type 2B and identifies the typical electrophoretic profile of vCJD resPrP^D^. *Statistically significant difference (p < 0.002) between adjacent T2B values.

### Histopathology and immunohistochemistry

**Figure 1:**
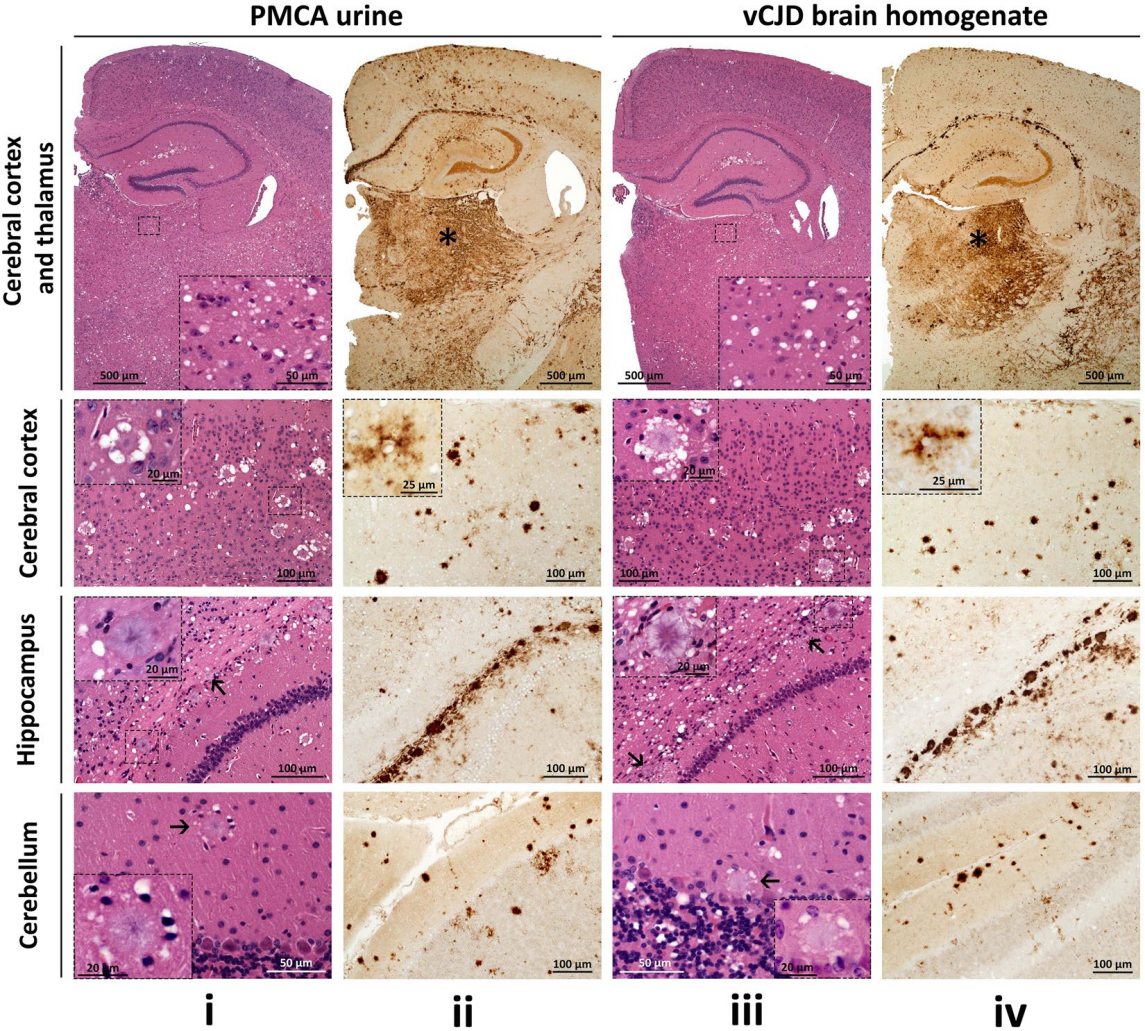
Histopathology and PrP immunohistochemistry of brain regions from Tg40 mice. Hematoxylin and eosin (H.E.) staining (i and iii) and PrP immunohistochemistry (ii and iv). i and iii, 1^st^ row: Prominent spongiform degeneration (SD) in subcortical regions. Insets: SD of the thalamus. 2^nd^ row: Typical florid plaques characterized by a dense eosinophilic core and a ring of surrounding SD vacuoles. Insets: individual florid plaques. 3^rd^ row: Non-florid plaques of different sizes at the border between the alveus of the hippocampus and the corpus callosum. Arrows: small plaques. Insets: Non-florid plaques. 4^th^ row: Florid plaques (arrow) populated the molecular layer of the cerebellum. Insets: florid plaques. ii and iv, 1^st^ row: PrP immunostaining of cortical and subcortical regions. PrP immunostaining is particularly intense in the thalamus (asterisk). 2^nd^ row: Intense PrP immunostaining of the florid plaques. Insets: peri-cellular PrP immunostaining (“stellate cells”). 3^rd^ row: Non-florid PrP plaques and loose PrP aggregates distributed along the border between the hippocampal alveus and the corpus callosum. 4^th^ row: PrP plaques and plaque-like PrP aggregates located in the molecular and granular layers of the cerebellum. PrP monoclonal antibody 3F4.

The histopathological features of the Tg40 mice challenged with PMCA-treated urine essentially reproduced type and distribution of the lesions associated with the inoculation of vCJD BH. In both conditions, spongiform degeneration (SD) consisted mostly of large vacuoles that predominantly affected subcortical formations including thalamus, hypothalamus, basal ganglia and brain stem (Figs 1i, iii, row l, and 2A). By contrast, florid plaques (FP) populated mostly the cerebral cortex and, to a lesser extent, subcortical regions and cerebellum (Fig. 1i, iii, rows 2 and 4). Non-FP plaques of various sizes were also seen often lined up along the remnants of the lateral ventricles above the hippocampus (Fig. 1i, iii, row 3).

Diffuse PrP immunostaining codistributed with the SD, with intensities that appeared to match the SD severity (Fig. 1ii, iv, row 1). FP and non-FP were strongly immunostained while stellate apparently pericellular PrP deposits were occasionally seen in the cerebral cortex (Fig. 1ii, iv, rows 2 and 3). PrP immunostaining affected also the cerebellum, including the molecular layer and, occasionally, the granular layer and the deep white matter (Fig. 1ii, iv, row 4).

**Figure 2:**
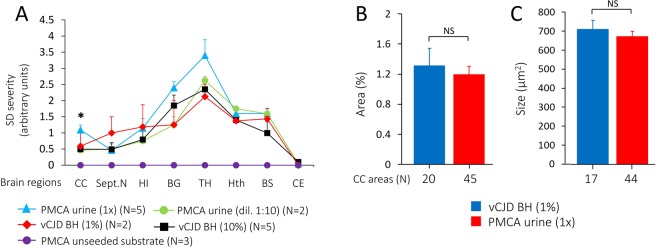
Lesion profiles and density of florid plaques in brains of Tg40 mice inoculated with PMCA-treated urine or brain homogenate (BH) from vCJD. (**A**) The profiles of topographic distribution and severity of SD in each group of mice were similar; subcortical regions were predominantly affected with the thalamus showing the most severe lesions. Inoculation of undiluted (1x) PMCA-treated urine resulted in widespread increase of SD which was significantly more severe in the cerebral cortex when compared with the SD in mice inoculated with 10% BH (*p < 0.03). No lesions were detected in mice inoculated with PMCA-unseeded substrate. CC: Cerebral cortex; Sept.N: Septal nuclei; HI: Hippocampus; BG: Basal ganglia; TH: Thalamus; Hth: Hypothalamus; BS: Brainstem; CE: Cerebellum. (**B**) Florid plaque burden, expressed as the percentage of the area occupied by the florid plaques, was comparable in the two groups of mice. (**C**) Florid plaque size, expressed in µm^2^. In **B** and **C** values were obtained from the examination of all available CC areas. Similar values were obtained when equal number of areas were compared in the two groups (data not shown). All data are expressed as mean ± SEM. NS: not significant.

Lesion profiles showed an overall similar distribution of lesions in Tg mice inoculated with PMCA-treated urine or vCJD BH. However, lesions were slightly more severe in mice challenged with 1x PMCA-treated urine than in those receiving vCJD BH (1% or 10%) or 1:10 diluted PMCA-treated urine (Fig. 2A). Neither prion-related lesions nor PrP^D^ deposits were observed in Tg40 mice inoculated with PMCA-unseeded substrate and raw vCJD urine up to 673 dpi and 707 dpi, respectively (Fig. 2A and data not shown). Morphometric analysis of the density and size of FP in the cerebral cortex revealed no significant differences between the two groups of inoculated mice (Fig. 2B,C).

### PrP^D^ characterization

**Figure 3:**
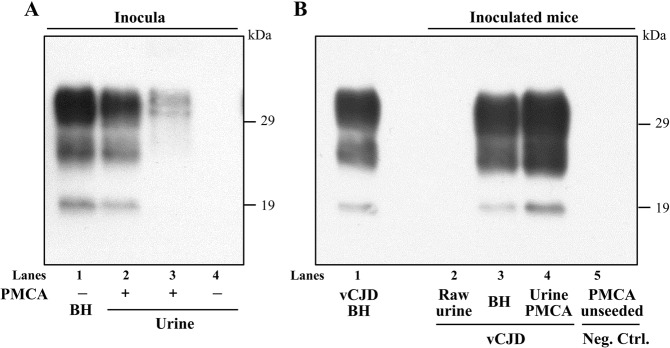
Immunoblots of PK-resistant PrP^D^ (resPrP^D^) from Tg40 mice inoculated with PMCA-treated vCJD urine and vCJD BH along with native inocula and negative controls. All detected resPrP^D^ species examined showed the typical T2B profile characterized by the 19 kDa unglycosylated band identifying resPrP^D^ type 2, and the overrepresentation of the diglycosylated form. (**A**) resPrP^D^ was detectable in PMCA-treated urine but not in raw urine. Undiluted PMCA-treated urine were loaded 20 or 2 times more than vCJD BH 10% in lanes 2 and 3, respectively. In lane 4 is PMCA-untreated and concentrated urine (1 ml equivalent) after PK digestion. (**B**) No resPrP^D^ was detected in Tg mice inoculated with raw (non-PMCA-treated) or with unseeded PMCA substrate. BH from Tg40 inoculated with 10% vCJD BH or undiluted PMCA-treated urine (lanes 3 and 4) were loaded 10 times more than vCJD BH 10% (lane 1). Neg. Ctrl.: negative control; inoc.: inoculum. Ab: 3F4.

Regardless of the inoculum, Tg40 animals injected with PMCA-amplified vCJD urine or with untreated vCJD brain homogenate showed the presence of a PK-resistant PrP^D^ (resPrP^D^) displaying the same electrophoretic profile, which was characterized by the prevalence of the diglycosylated band and the mobility to 19 kDa of the unglycosylated isoform (type 2B) (Fig. 3). resPrP^D^ representation in undiluted PMCA-treated urine was approximately 20 times lower than that of vCJD BH (10%) (Fig. 3). No resPrP^D^ was detected in Tg40 mice inoculated with raw vCJD urine (Fig. 3).

**Figure 4:**
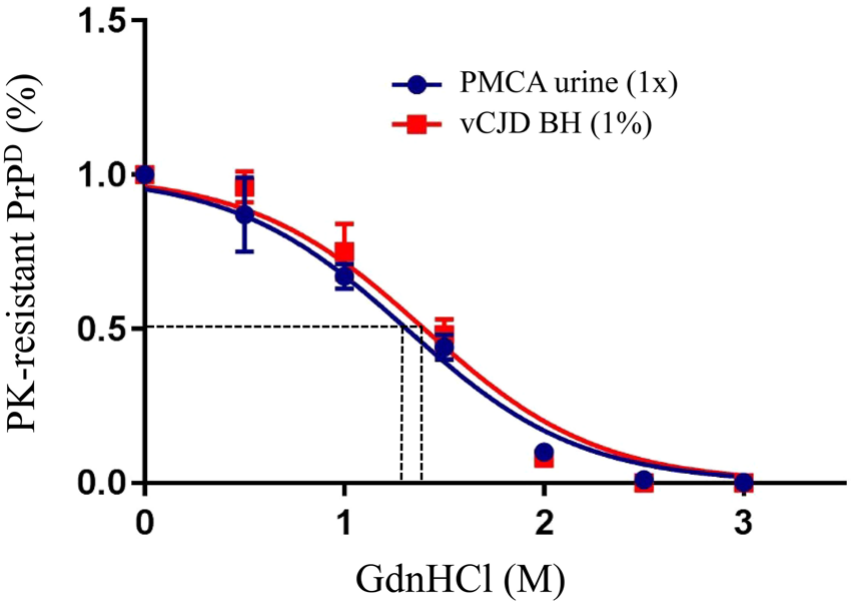
Conformational stability immunoassay of resPrP^D^ from brain of Tg40 inoculated with undiluted PMCA-treated vCJD urine or 1% vCJD BH revealed no statistically significant difference between curves depicting the rate of conversion of resPrP^D^ to PK-sensitive PrP as a function of the amount of GdnHCl. The guanidine concentrations needed to render half of the PrP^D^ sensitive to PK, [GdnHCl]_1/2_, were 1.31 ± 0.1 M and 1.4 ± 0.1 M, respectively (N = 3 for each group).

The stability of resPrP^D^ species extracted from Tg40 mice inoculated with either PMCA-treated vCJD urine or vCJD BH was tested with the conformational stability immunoassay (CSI). No significant difference was detected between the two resPrP^D^ species in their rate of denaturation following treatment with increasing amounts of the denaturant guanidine hydrochloride (GdnHCl). When the GdnHCl treatment was expressed as GdnHCl molar quantity reducing the amount of resPrP^D^ to half ([GdnHCl]_1/2_), the values obtained were 1.31 ± 0.1 M and 1.4 ± 0.1 M (Fig. 4).

## Discussion

We have recently shown that urine of patients affected by vCJD harbors minute amounts of PrP^D^ estimated at 1 × 10^−16^ g/mL, which is approximately 12 orders of magnitude smaller than the PrP^D^ amount in the vCJD brain tissue (1 × 10^−4^ g/g)^11^. Despite the small amount, urine PrP^D^ can be used to diagnose the presence of prion disease in vCJD with 93% sensitivity and 100% specificity^11^. This low PrP^D^ content is consistent with our finding that none of the 96 Tg40 mice inoculated with raw urine formed detectable resPrP^D^ especially considering that at least in sCJD the Tg40 limit detectability of brain PrP^D^ is approximately 1 × 10^−5^ equivalent concentration^14^.

The complex physiology of urine formation raises the question of how and at which stage PrP^D^ spreads to urine. Although the precise mechanism remains unidentified, three propagation processes have recently been considered^12^. Given its significant presence in vCJD blood, PrP^D^ may invade urine during the stage of glomerular filtration, tubular secretion or both. Indeed, increase PrP^D^ representation in urine has been observed experimentally in the presence of kidney inflammatory processes associated with increase of urine protein excretion^15^. Urine contains relatively high amounts of PrP that is truncated at the N-terminus and harbors an incomplete glycosylphosphatidylinositol anchor suggesting that it is shed from cell surfaces^16^. However, this truncated PrP has been identified as C-terminal fragment C1^16^, a product of normal PrP^C^ metabolism that is an unlikely substrate for conversion to PrP^D^ since the N-terminal cleavage breaks the 100–130 residue domain considered important for the conversion of PrP^C^ to PrP^D^^17–21^. It is also noteworthy in this context that contrary to many visceral organs, no PrP^D^ has been demonstrated in the bladder^9^. Finally, urine PrP^D^ might result from traditional contiguous PrP^D^ propagation to urine from kidney, blood or both especially at the level of the nephrons^9,10,12^.

In the present study, we show that PMCA-treated urine PrP^D^ is competent to transmit to Tg mice expressing human PrP a prion disease that, as to the histopathological phenotype and resPrP^D^ properties, is indistinguishable from the disease transmitted by inoculation with vCJD BH. These findings suggest that urine resPrP^D^ preserves the strain characteristics of the brain resPrP^D^. Therefore, the mechanism of urine invasion by PrP, whatever that might be, as well as the PMCA procedure, must preserve the conformational characteristics of brain-generated resPrP^D^ in vCJD. The present study also confirms the competence of PMCA to accurately replicate PrP^D^ strain features enciphered in the original seed. This PMCA property, originally reported for the 263K hamster scrapie agent^22–24^, is here demonstrated for the first time in a human prion strain. An additional corollary to the present finding is that the non-invasive PMCA-based urine diagnostic test may be used reliably to determine not only the presence of prion disease but also to establish the diagnosis of vCJD.

Presence of PrP^D^ in urine has been reported in 40% of patients affected with various subtypes of sCJD, by far the most prevalent type of human prion diseases^1^, using a solid-state binding matrix to capture and concentrate urine PrP^D12^. By contrast, no PrP^D^ signal was detected in sCJD urine following the same experimental conditions as those used for PMCA analysis of vCJD urine samples^11^. It has been reported that PrP^D^ associated with sCJDMM1, the most common subtype of sCJD, is a very inefficient seed for PMCA when using the same conditions described in this article (Camacho *et al*., Prion 2017; May 23–26, 2017; Edinburgh, UK). Finding a solution to this technical hurdle may afford a urine-based diagnostic test that, along with codon 129 PrP genotype determination, may provide the diagnosis of sCJD subtype.

## Materials and Methods

### Biological Safety

Prion material was handled in a biosafety level 2 (BSL-2) laboratory and all mice were housed in a BSL-2 room of Case Western Reserve University animal facility.

### Urine samples

Urine samples were obtained from the National Prion Disease Pathology Surveillance Center (NPDPSC). Samples were collected, during the symptomatic period, from a vCJD patient, who died at 25 years of age, 32 months after clinical onset^9,25^. Urine samples were stored at −80 °C until use.

### Protein misfolding cyclic amplification (PMCA)

PMCA of vCJD urine and unseeded substrate was carried out as previously reported^11^. Briefly, 1 ml of urine was centrifuged at low speed (5000 × *g* for 20 minutes at 4 °C) to separate debris. Two fractions, supernatant (S1) and pellet (P1), were obtained. S1 was then centrifuged at high speed (100,000 × *g* for 1 hour at 4 °C) and the pellet (P2a) directly resuspended in 10% homogenate (BH) prepared from brains of transgenic (Tg) mice expressing human PrP-129M (Tg line with PMCA efficiency higher than that of Tg40), which also served as substrate for PMCA. P1, containing cellular debris, was resuspended in 1 ml of water and subjected to high-speed centrifugation (100,000 × *g* for 1 hour at 4 °C). The pellet (P2b) was resuspended in Tg mice-BH, and identified as P2a. The two resuspended pellets (P2a and P2b) were pooled and processed through PMCA cycles. After 96 cycles, the Tg mouse PrP^C^ substrate was refreshed, and sample subjected to a new round of PMCA cycles. The final sample was obtained after 6 rounds of PMCA. The same Tg mouse BH used as a substrate, left unseeded and similarly treated with 6 rounds of PMCA served as negative control.

### Transgenic mice and inoculations

Tg mice expressing wild type level human PrP-129M in mouse PrP null background (Tg40), were used for the transmission study^26^. One hundred thirty four Tg40 mice received intracerebral inoculation (30 µl) of the following samples: (i) PMCA-treated vCJD urine undiluted (1x) or diluted 1:10 (9 and 8 mice, respectively); (iii) vCJD-BH from the same urine donor at 10% or 1% w/v concentration (9 and 3 mice, respectively); (iii) substrate subjected to PMCA without being supplemented with vCJD-urine samples as negative control (9 mice), and (iv) raw vCJD urine (96 mice).

### Histopathology, immunohistochemistry, lesion profiles and morphometric analysis

Mouse brains were harvested and bisected along the inter-hemispheric fissure. One half was stored at −80 °C until needed for biochemical studies; the other was fixed in formalin and used for histopathological and immunohistochemical examinations. Four coronal slices, obtained from the formalin-fixed half brain, at approximately bregma 0.5 mm, −1.7 mm, −3.8 mm and −6.0 mm, were processed for microscopic examination following staining with hematoxylin and eosin (H.E.) or immunostaining with monoclonal antibody (mAb) 3F4 as previously described^27,28^.

Lesion profiles were generated by semi-quantitative evaluation of the severity of spongiform degeneration (SD) on a 0–4 scale, in eight brain regions examined on H&E stained sections (also see Fig. 1). Morphometric analysis to assess density of florid plaques was performed as previously described^29^. Briefly, morphometric analysis was carried out on dorsal, intermediate and ventral cerebral cortices at the three anterior bregma levels. Density was expressed as percentage of the cortical area occupied by florid plaques, whereas size was expressed in µm^2^, and calculated by dividing the area occupied by the florid plaques for their number. To obtain a comparable sample size, the random function (Excel software) was used to randomly select areas of cerebral cortex.

### Western blot

Ten percent BH, (10% w/v) were prepared on ice in PBS Sarkosyl 2% and aliquots digested with 20U/ml PK (corresponding to 400 µg/ml when PK specific activity is 50U/mg) (Roche Diagnostics) for 1 h at 37 °C. 1 ml of PMCA-untreated vCJD-urine was centrifuged at high speed (100,000 × *g* for 1 hour at 4 °C) and PK digestion (5U/ml, corresponding to 100 µg/ml when PK specific activity is 50U/mg), was performed for 1 h at 37 °C on the pellet resuspended in 10 µl of PBS Sarkosyl 2%. PK digested. The PK digested samples were diluted in sample buffer (final concentration: 3% SDS, 4% β-mercaptoethanol, 10% glycerol, 2 mM EDTA, 62.5 mM Tris, pH 6.8) and boiled for 8 min before loading. Protein samples were separated with Tris-Glycine SDS-PAGE in 15% Criterion Tris-HCl polyacrylamide precast gels (Bio-Rad Laboratories, Hercules, CA, USA) and transferred to Immobilon-P PVDF transfer membrane (EMD-Millipore, Billerica, MA, USA) for 2 h at 60 V, blocked with 5% nonfat dry milk in 0.1% Tween 20-Tris-buffered saline, pH 7.5, and probed with the monoclonal anti-PrP antibody 3F4^27^. The immunoreactivity was visualized by enhanced chemiluminescence (Pierce ECL 2, Fisher Scientific, Hampton, NH, USA) on Kodak BioMax Light films (Eastman Kodak Co., Rochester, NY, USA).

### Conformational stability immunoassay (CSI)

CSI was performed as previously described^30–32^ with minor modifications. Aliquots of 10% BH in LB100 pH 8.0 (100 mM Tris HCl pH 8.0, 100 mM NaCl, 0.5% Nonidet P-40, 0.5% sodium deoxycholate, 10 mM EDTA) were centrifuged for 10 min at 1,000 × g at 4 °C and pellets discarded. 100 µl of the supernatants were diluted with an equal volume of GdnHCl to obtain a final concentration ranging from 0 to 4 M and incubated for 1.5 h at room temperature. GdnHCl was subsequently removed by precipitation with 5 volumes of methanol with overnight incubation at −20 °C and centrifuged for 30 min at 18,000 × g. Pellets were resuspended in 100 µl LB 100 pH 8.0 by sonication. Each aliquot was digested with 5U/ml PK for 1 h at 37 °C. The reaction was stopped by addition of 2 mM PMSF. After denaturation, samples were analyzed by WB (Ab: 3F4) and developed by Odyssey Classic infrared imaging system (LI-COR Biosciences). PrP densitometry was performed by Odyssey V3.0 software (LI-COR Biosciences). After normalization, the data were plotted and expressed as mean ± standard deviation. Conformational stability, evaluated as a function of resPrP^D^ conversion to PK-sensitive PrP following exposure to increasing concentrations of GdnHCl, was best fitted to a sigmoidal dose-response equation by GraphPad Prism 7 (GraphPad Software Inc.). The formula [GdnHCl]_1/2_ ± SD was calculated for the two groups and compared by statistical analysis.

### Statistical analysis

Statistical analyses were performed with (i) Student’s T-test, for lesion profile, florid plaque density-size analyses, and conformational stability immunoassay, and (ii) analysis of variance (One-Way ANOVA) followed by Tukey’s multiple comparison test, for incubation periods (days post inoculation, dpi).

### Ethics statement

The human samples used in the current study were provided by NPDPSC. Written informed consent for research was obtained from the patient according to the Declaration of Helsinki. No minor participants were included in the study. All procedures were performed under protocols approved by the Institutional Review Board for Human Investigation of University Hospitals, Case Medical Center of Cleveland, OH, and as per regulations of the Declaration of Helsinki. All animal studies were carried out in accordance with the recommendations in the Guide for the Care and Use of Laboratory Animals of the National Institutes of Health. The protocols were approved by the Institutional Animal Care and Use Committee of the Case Western Reserve University (Protocol number 2012-0199).

## Data Availability

All data generated or analysed during this study are included in this published article.
